# Genetic and transcriptomic analyses provide new insights on the early antiviral response to VHSV in resistant and susceptible rainbow trout

**DOI:** 10.1186/s12864-018-4860-1

**Published:** 2018-06-19

**Authors:** Eloi R. Verrier, Carine Genet, Denis Laloë, Florence Jaffrezic, Andrea Rau, Diane Esquerre, Nicolas Dechamp, Céline Ciobotaru, Caroline Hervet, Francine Krieg, Luc Jouneau, Christophe Klopp, Edwige Quillet, Pierre Boudinot

**Affiliations:** 1 VIM, INRA, Université Paris-Saclay, 78350 Jouy-en-Josas, France; 2 GABI, INRA, AgroParisTech, Université Paris-Saclay, 78350 Jouy-en-Josas, France; 3 GenPhySE, INRA, Université de Toulouse INPT ENSAT, Université de Toulouse INPT ENVT, 52627 Castanet-Tolosan, France; 4 Plateforme Bioinformatique Toulouse, Midi-Pyrénées UBIA, INRA, 52627 Castanet-Tolosan, France; 5 Present address: Inserm, Institut de Recherche sur les Maladies Virales et Hépatiques UMRS1110, Université de Strasbourg, F-67000 Strasbourg, France; 6 Present address: GenPhySE, INRA, Université de Toulouse INPT ENSAT, Université de Toulouse INPT ENVT, 52627 Castanet-Tolosan, France; 7 Present address: BioEpAR, INRA, Oniris, 44307 Nantes, France

**Keywords:** Rainbow trout, VHSV, Antiviral resistance, Transcriptome deep sequencing, QTL

## Abstract

**Background:**

The viral hemorrhagic septicemia virus (VHSV) is a major threat for salmonid farming and for wild fish populations worldwide. Previous studies have highlighted the importance of innate factors regulated by a major quantitative trait locus (QTL) for the natural resistance to waterborne VHSV infection in rainbow trout. The aim of this study was to analyze the early transcriptomic response to VHSV inoculation in cell lines derived from previously described resistant and susceptible homozygous isogenic lines of rainbow trout to obtain insights into the molecular mechanisms responsible for the resistance to the viral infection.

**Results:**

We first confirmed the presence of the major QTL in a backcross involving a highly resistant fish isogenic line (B57) and a highly susceptible one (A22), and were able to define the confidence interval of the QTL and to identify its precise position. We extended the definition of the QTL since it controls not only resistance to waterborne infection but also the kinetics of mortality after intra-peritoneal injection. Deep sequencing of the transcriptome of B57 and A22 derived cell lines exposed to inactivated VHSV showed a stronger response to virus inoculation in the resistant background. In line with our previous observations, an early and strong induction of interferon and interferon-stimulated genes was correlated with the resistance to VHSV, highlighting the major role of innate immune factors in natural trout resistance to the virus. Interestingly, major factors of the antiviral innate immunity were much more expressed in naive B57 cells compared to naive A22 cells, which likely contributes to the ability of B57 to mount a fast antiviral response after viral infection. These observations were further extended by the identification of several innate immune-related genes localized close to the QTL area on the rainbow trout genome.

**Conclusions:**

Taken together, our results improve our knowledge in virus-host interactions in vertebrates and provide novel insights in the molecular mechanisms explaining the resistance to VHSV in rainbow trout. Our data also provide a collection of potential markers for resistance and susceptibility of rainbow trout to VHSV infection.

**Electronic supplementary material:**

The online version of this article (10.1186/s12864-018-4860-1) contains supplementary material, which is available to authorized users.

## Background

Viral outbreaks are a major global problem for aquaculture industry and fisheries, with important economic consequences [1, 2]. Novirhabdovirus are responsible for severe hemorrhagic diseases with significant fish mortality [3]. VHSV (viral hemorrhagic septicemia virus) infects farmed rainbow trout with severe consequences since no treatment or prophylactics allows to cure or prevent these major virus infections [4]. It also causes outbreaks in a number of wild finfish species in fresh and marine water worldwide [5] and is listed as a notifiable disease in many nations and international organizations [6]. VHSV is a single-stranded RNA virus containing a non-segmented RNA genome of approximately 12,000 nucleotides (nt) coding for five structural proteins and a sixth functional cistron which codes for a non-structural protein (NV), a feature specific of novirhabdoviridae. In rainbow trout, a high heritability of resistance to VHSH (from 0.57 to 0.63) has been reported in two independent studies [7, 8]. In the same line, a wide range of susceptibility to VHSV has been reported, with the existence of fish lines fully resistant to waterborne infection [9], suggesting that host genetics plays a key role in virus susceptibility. Several lines of evidence suggest that rainbow trout VHSV resistance implicates innate mechanisms. First, the correlation between fish survival and virus load in infected fin explants [10, 11] strongly suggests the contribution of innate or intrinsic virus resistance cellular factors. Another striking evidence was the exquisite correlation between both the resistance to waterborne VHSV infection of rainbow trout isogenic lines and of the fibroblast-like cell lines derived from these fish [12]. Finally, the identification in resistant x susceptible crossed trout families of a major genomic region Quantitative Trait Loci (QTL) controlling both fish survival and viral growth in fin tissue which further supports the crucial role of innate or intrinsic VHSV resistance factors [13]. Interestingly, the differences observed in the type-I interferon (IFN) induction between resistant and susceptible cell lines suggests that an early activation of the innate antiviral response is well correlated with the resistance to infection [12]. However, the detailed mechanisms of antiviral resistance remain to be determined. As in mammals, type-I IFN are responsible for the induction of a high number of genes (Interferon Stimulated genes, ISG) after virus infection in fish and especially in rainbow trout, including conserved antiviral genes such as *rsad2/viperin* [14], *mxa* [15], and *isg15* [16, 17], which constitute the first cellular line of defense against viral infection [18–21]. A differential induction of ISG based on genetic background, in kinetics or amplitude, may explain variation in the outcome of a viral infection. In this context, a comparison of transcriptome responses at an early stage of VHSV infection between resistant and susceptible fish would help to understand the resistance mechanisms.

With the recent development of next-generation sequencing, an increasing number of high-throughput transcriptomic studies allowed the description of fish response to virus infection [22–26]. Interestingly, transcriptomic analyses in resistant and susceptible Atlantic salmon (*Salmo salar*) after infectious pancreatic necrosis virus (IPNV) infection highlights significant differences in the immune responses to infection [27].

In this study, we aimed to gain new insights on the VHSV infection response mechanisms of cells from fish with resistant and susceptible genetic backgrounds. To do so, we took advantage of two rainbow trout homozygous isogenic lines exhibiting highly contrasted levels of susceptibility to virus infection: the resistant line B57 and the susceptible line A22 [9]. Using B57 and A22 derived offspring infected with VHSV, we first confirmed through a genetic study that the major QTL, which we previously identified on rainbow trout chromosome 3 (RT31 linkage group) [13], was effectively present in these genetic backgrounds. Using B57 and A22 derived fibroblastic cell lines, respectively resistant and susceptible as fish from which they derived, we therefore performed a genome-wide characterization of the transcriptome response to the virus through RNA deep sequencing. Both cells responded to the stimulation, but the activation of the type I IFN pathway was more intense in resistant cells, with many classical ISGs upregulated in the B57 cell line, but not in A22. Moreover, we showed that a number of key factors of type-I IFN pathway were more expressed in B57 cells than in A22 cells at the basal level, which likely contributes to the difference of resistance.

Collectively, our results pave the way for further studies to characterize the antiviral mechanisms mediating the protection and provide an array of potential key genes involved in the resistance to VHSV orchestrated by the major QTL previously reported.

## Results

### A major QTL controls the differences of resistance to VHSV in B57 and A22 fish

**Fig. 1:**
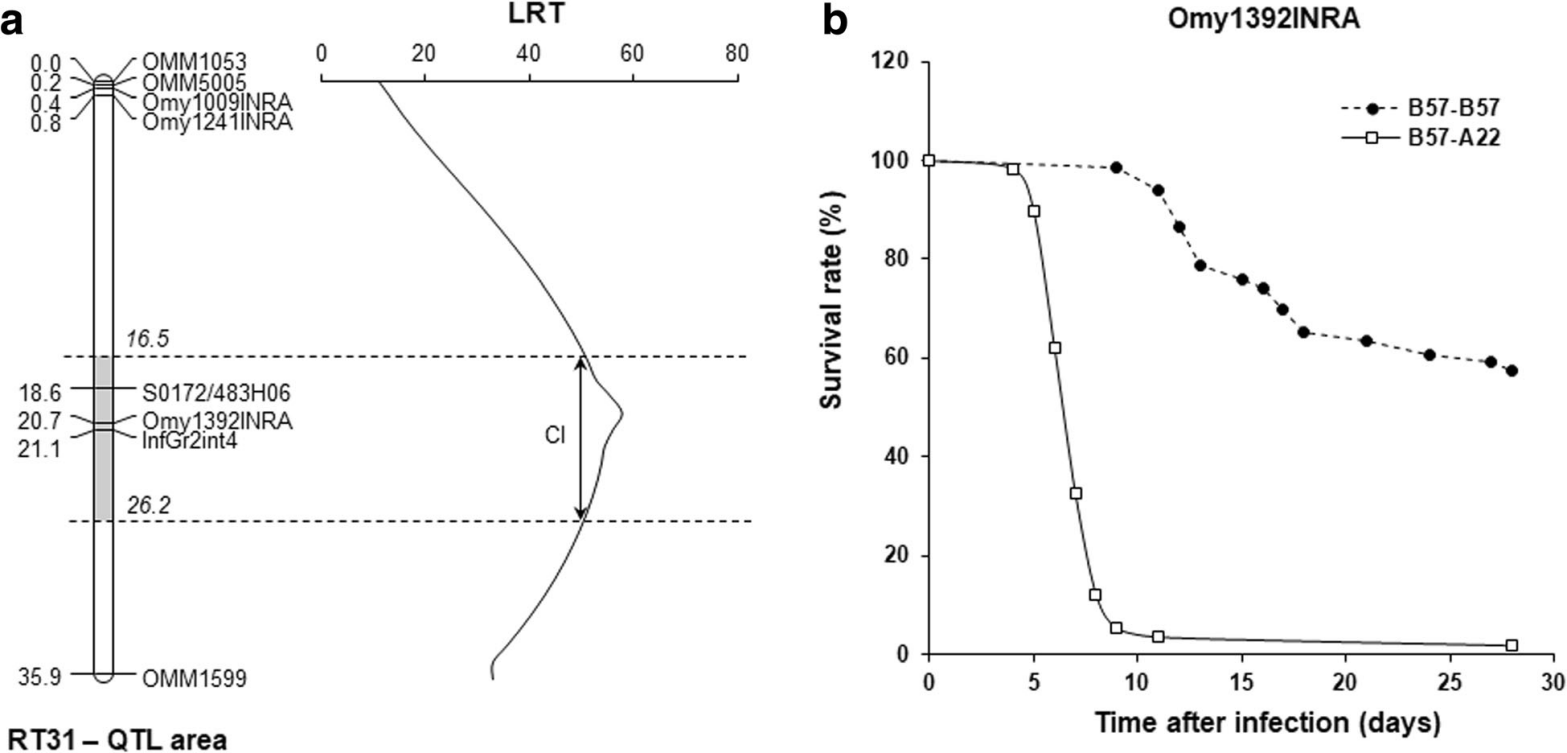
Detection of the major QTL associated with resistance to VHSV on Omy3 (linkage group RT31) in a B57-A22 backcross. **a** Likelihood ratio profiles for survival associated QTL on Omy3 in a B57-A22 backcross. Two aquaria of 119 and 120 fish from a B57-A22 backcross were infected with VHSV 07–71 (waterborne challenge). A total of 8 markers localized in the previously identified QTL area on Omy3 were genotyped. Genetic distance (cM) between the markers is indicated on the left. Likelihood (LRT) values were obtained using the QTLMap software. The 95% confidence interval (CI) of the QTL was obtained using the Hendge Li method and is indicated in grey. **b** Conservation of the effect of the QTL on survival rate in BCB57-A22 backcross progeny after infection with VHSV 07–71 by injection. 125 fish from a B57-A22 backcross were injected with VHSV. Mortality was monitored daily and surviving fish were sacrificed at day 28 post infection. The allelic status at Omy1392INRA marker was determined for every fish, and cumulative death curves were built for the two alternative genotypes (B57-A22 and B57-B57)

To understand the mechanisms underlying the differences of susceptibility to VHSV between B57 and A22 isogenic lines, we first looked for the presence of the major QTL we previously identified [13]. To do so, F2 segregating offspring were produced by backcrossing a B57-A22 F1 hybrid and a parental B57 (BC57-A22). BC57-A22 offspring were then waterborne challenged with VHSV 07–71, and individuals were genotyped for 8 markers (microsatellites or SNP) belonging to the major QTL area on chromosome Omy3 (RT31 linkage group). The presence of a QTL for resistance in the region was then tested using an interval mapping method implemented in the QTLMap software. As shown in Fig. 1a, a highly significant QTL was found in this area, confirming the key role of this genomic region in the difference of resistance to VHSV among lines B57 (resistant) and A22 (susceptible). Moreover, our results allow to map the major QTL more precisely, most likely between S0172/483H06 and Omy1392INRA markers within a limited area as defined by the interval of confidence. Notably, 27% of the phenotypic variance of the time to death after infection was explained by the genotype of challenged fish at Omy1392INRA marker (*p* < 0.001). To go deeper into its characterization, we aimed to determine whether the QTL was observed whatever the route of infection. To answer this question, 125 BC57-A22 offspring were injected intraperitoneally with VHSV (strain 07–71), and individuals were genotyped for Omy1392INRA, one of the flanking markers of the most likely position of the QTL. Survival curves were then determined according to the allelic status at Omy1392INRA (124 genotypes out of 125 individuals were clearly determined). Among the 41 surviving fish at the end of the challenge, 39 were homozygous for B57 allele (resistant) at Omy1392INRA (B57-B57), when 56 out of 83 dead fish were heterozygous (B57-A22) at the marker (χ^2^ = 43.3; *p* < 0.001). Moreover, the kinetics of mortality was dramatically faster in the heterozygous progeny (Fig. 1b), suggesting a dominant susceptibility effect.

This result highlights the importance of this genomic region for the resistance to VHSV whatever the route of infection (waterborne or via intraperitoneal injection). Also, they indicate that A22 and B57 lines constitute a relevant pair of genetic backgrounds to understand the nature of genetic resistance to VHSV in rainbow trout.

### Transcriptional response of A22 and B57 cells induced by inactivated VHSV

**Fig. 2:**
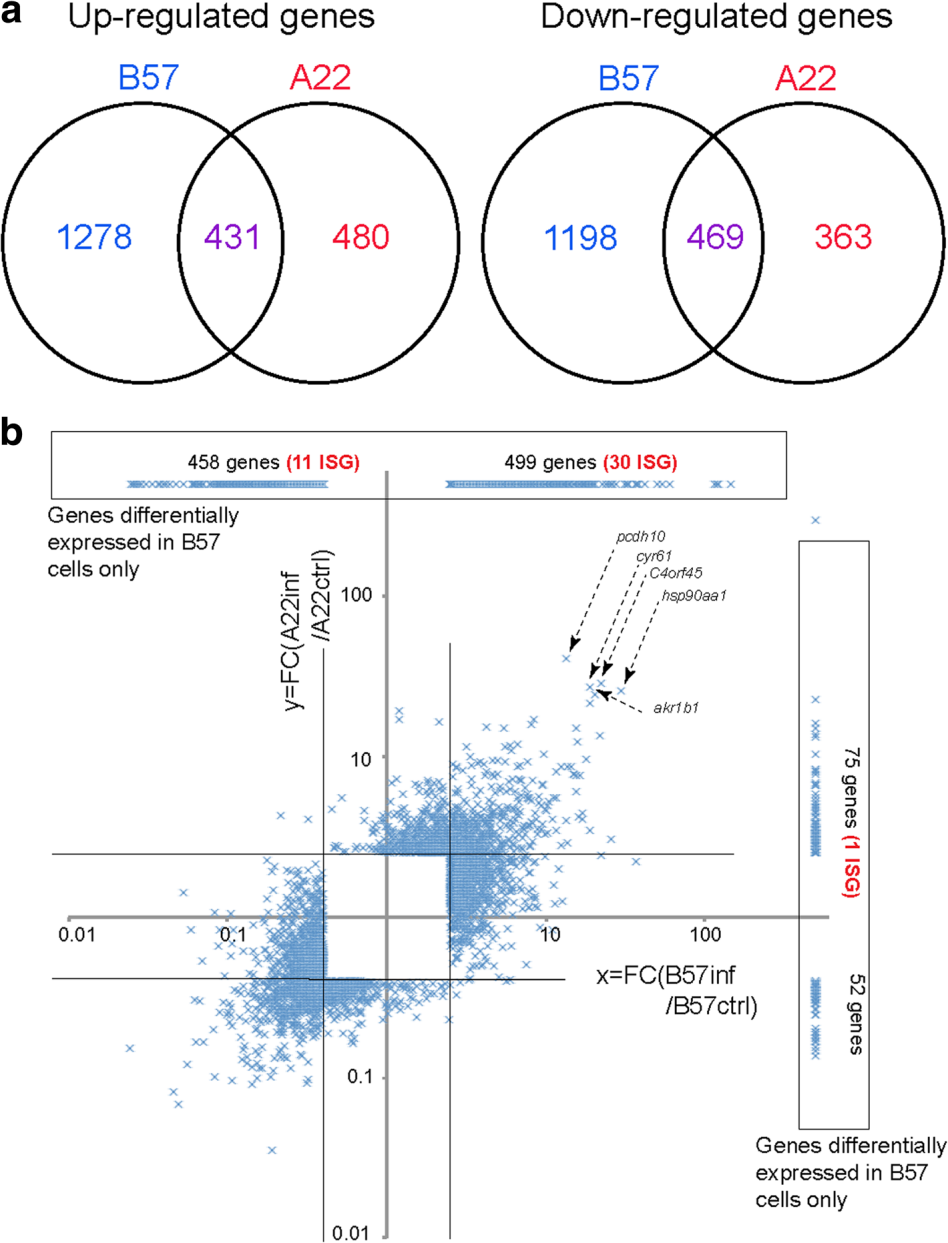
Global analysis of RNAseq data. **a** Venn diagram showing the number of genes significantly induced (respectively repressed) in A22 or B57 cells (*p* < 0.01; Fold Change (FC > 2.5 or < 0.4) by inactivated VHSV: A22ind and B57ind (respectively A22rep and B57rep). Numbers of genes modulated in A22 only are in red, numbers of genes modulated only in B57 are in Blue, and numbers of genes modulated in both are in purple; numbers in categories empty by definition are in grey background. **b** LogFC/logFC representation. Average FC values are represented for genes significantly modulated (*p* < 0.01; Fold Change (FC > 2.5 or < 0.4) by inactivated VHSV. The FC of genes differentially expressed in only one cell line were represented on a line set up at an arbitrary level of 100 (induced genes) or 0.01 (repressed genes) on the axis of the other, for a clear representation of the data. The numbers of such genes are indicated in the figure. Genes with a homolog in the list considered by Schoggins et al. [29] were counted as ISG, as in Fig. 3

To understand the early transcriptomic differences in response to VHSV in resistant and susceptible isogenic lines, B57 and A22 derived cell lines were inoculated with inactivated VHSV (07–71 strain), which induces a significant type I IFN response even if it may not exactly recapitulate the infection with the live VHSV. Using inactivated virus limited the biases of stimulation when comparing A22 and B57 transcriptome responses. Indeed, infection with active VHSV would induce a substantial accumulation of viral RNA in A22 cells (but not B57 cells), which may saturate the viral sensing system [12]. In contrast, the inactivated virus led to a comparable exposure of A22 and B57 cells to viral stimulation, and avoided heavy contamination of the transcriptome by viral RNA. Gene expression was analyzed by RNAseq 24 h after virus inoculation: differentially expressed genes between cells incubated with the virus and controls were determined for each line, then compared across genetic backgrounds. This time point was selected based on our previous analysis of the type I IFN response in A22 and B57 cells, to warrant the detection of a potential contrast between the responses in the two genetic backgrounds. We have previously observed that the type I IFN response to VHSV infection was much faster in B57 cells, with mRNA for the IFNφ1 (aka IFN1) already expressed at 4 h post infection (hpi), while it was just detectable in A22 cells only at 8hpi. To observe a well-developed response we therefore characterized the transcriptome of both cell lines 24 h after incubation with the inactivated virus. Differentially expressed genes between cells incubated with the virus and controls were determined for each line, then compared across genetic backgrounds. Almost twice as many genes were modulated by the inactivated virus in the B57 cell line (3380 genes), compared to the A22 cell line (1747 genes) (Fig. 2a; Additional file 1). About 50% of modulated genes were respectively up- and down regulated in each genetic background: 1711 up for 1669 down in B57, and 913 up for 834 down in A22. Importantly, only 431 and 469 genes were commonly up- (respectively down-) regulated in both cell lines, meaning that most of the transcriptional response was cell line specific. The stronger response in B57 cells was also demonstrated by higher fold changes of the genes modulated in B57 (and not in A22), compared to those modulated in A22 (and not in B57) (Fig. 2b, compare the line at the top of panel B with the column at the right side); additionally, 956 genes induced or repressed in B57 cells were not detected in A22 cells (28%), while only 126 differential genes in A22 were not expressed in B57 (7%). Overall, the dynamics of transcriptional changes across the basal gene expression level was not obviously different between A22 and B57 cells (Additional file 1). Taken together, these results suggest a much stronger response to virus inoculation in B57 cells compared to A22 cells.

### Strong induction of innate immune-related pathways in B57 following VHSV inoculation

To analyze the nature of the transcriptomic response to VHSV in both genetic backgrounds, we performed a functional annotation analysis mapping zebrafish homologs of rainbow trout modulated genes to zebrafish pathways in the KEGG database. As shown in Additional file 2, VHSV induced the modulation of metabolic pathways, such as sugar and lipid pathways, as well as global cellular signaling pathways, such as mTOR and MAPK. Interestingly, genes belonging to sugar metabolism were induced in both A22 and B57, in line with a previous observation that glycolytic pathways were induced after VHSV infection in zebrafish [28] (Table 1). Cell cycle-related pathways were found downregulated also in both cell lines (Table 2), which may reflect their common ability to slow cellular growth in response to virus infection.

**Table 1:** DR pathways induced in trout cell lines after inoculation with VHSV

Induced pathways			
B57		A22	
*1711 RT sequences / 1029 DR genes*		*913 RT sequences / 580 DR genes*	
KEGG pathway	*p*value	KEGG pathway	*p*value
Glycerophospholipid metabolism	0.0001	Fructose and mannose metabolism	0.002
**Cytokine-cytokine receptor interaction**	**0.01**	Biosynthesis of unsaturated fatty acids	0.01
**Jak-STAT signaling pathway**	**0.05**	Progesterone-mediated oocyte maturation	0.01
Fructose and mannose metabolism	0.06	Sphingolipid metabolism	0.01
Endocytosis	0.06	Fatty acid elongation	0.03
MAPK signaling pathway	0.08	Glutathione metabolism	0.03
Metabolic pathways	0.098	Inositol phosphate metabolism	0.05

*DR Danio rerio*

*RT* rainbow trout

**Bold:** Pathways related to antiviral innate immune response

**Table 2:** DR pathways repressed in trout cell lines after inoculation with VHSV

Repressed pathways			
B57		A22	
*1669 RT sequences / 1004 DR genes*		*834 RT sequences / 532 DR genes*	
KEGG pathway	*p*value	KEGG pathway	*p*value
FoxO signaling pathway	0.001	p53 signaling pathway	0.0004
Notch signaling pathway	0.01	Cell cycle	0.0006
p53 signaling pathway	0.01	Herpes simplex infection	0.002
Peroxisome	0.02	Apoptosis	0.04
Cell cycle	0.02	Retinol metabolism	0.07
Propanoate metabolism	0.03	Glycosaminoglycan biosynthesis - CS / DS	0.096
Apoptosis	0.05		
Wnt signaling pathway	0.07		
TGF-beta signaling pathway	0.07		
Purine metabolism	0.08		

*DR Danio rerio*

*RT* rainbow trout

Interestingly, pathways related to innate immunity (Table 1) and GO terms (data not shown) linked to viral infection were significantly elicited only in B57 cells, comprising genes involved in the interaction between cytokines and their receptors, and genes involved in Jak-STAT signaling pathways. Importantly, the rainbow trout homolog of zebrafish *ifnphi1* gene was upregulated (FC = 3.9) in B57 and was not detected in A22, confirming an early activation of IFN response in B57 and not in A22, which likely contributes to the difference of susceptibility between the two cell lines [12].

### Cells with VHSV resistant background express a much stronger type I IFN response than susceptible A22 cells

**Fig. 3:**
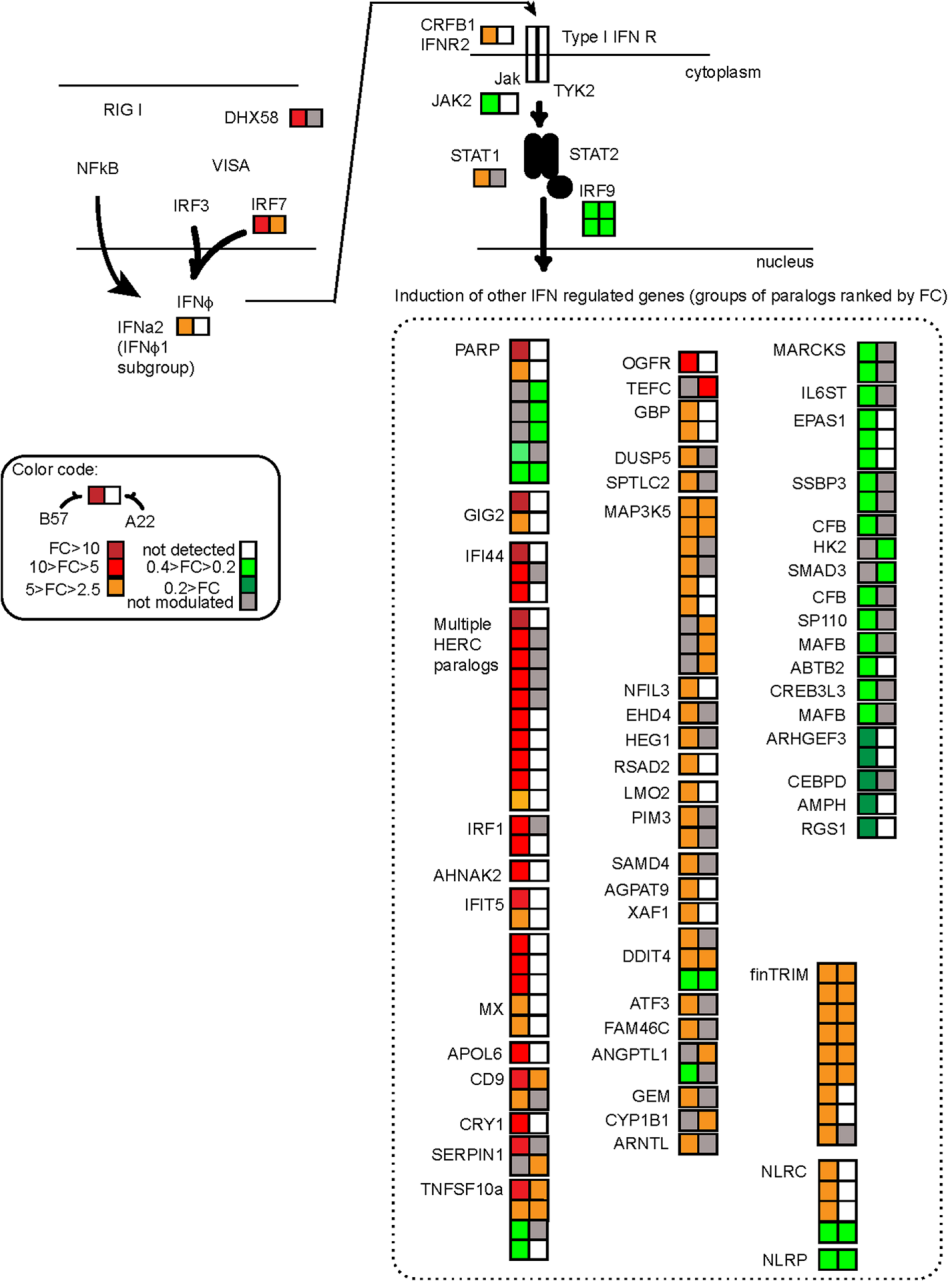
Type I IFN pathway. Analysis of gene expression responses related to the type I IFN pathway upon exposure of A22 and B57 cells to inactivated VHSV. The expression profiles at 24 h post-inoculation (infected versus control) were simultaneously mapped on the IFN pathway. Gene boxes are color coded according to FC as indicated. The pathway is based on knowledge of IFN signaling and ISG expression in mammalian and fish species. It is not meant to be exhaustive, and all the interactions between signaling components may not have been experimentally confirmed in rainbow trout (or even in fish)

Type I IFN pathway was an obvious target for a detailed comparison of the response of the two cell lines. To get a comprehensive overview of the ability of B57 to rapidly express ISGs after virus inoculation, we compared the type I IFN response triggered by the inactivated virus in B57 and A22 cells. To this purpose, we first mapped on the type I IFN pathway the expression of all trout genes having an ortholog in the list of human ISG used by Shoggins et al. in their systematic functional screen [29] (Additional file 3), together with genes that have been characterized as ISG in fish (Fig. 3). We also mapped the expression of the paralogs of these trout genes, since multiple copies due to whole genome duplication events during fish evolution have often complementary, related functions. Strikingly, most of the 78 ISG and related genes that were induced by the virus in B57 cells were not up-regulated or not even detected in A22 (i.e., 64 genes, see map of the IFN pathway in Fig. 3), including canonical markers of the type I IFN response such as *viperin/rsad2, isg15, ifi44, dhx58, ifit5* and *irf1*. In addition to these ISG homologs, fish specific ISG like *gig* [30], were up-regulated in B57 but not in A22 cells. Fourteen genes were induced in both B57 and A22 (but generally more induced in B57) and only 7 genes were induced in A22 only. Interestingly, the *crfb1* gene encoding the type I IFN receptor was up-regulated in B57 only (Fig. 3; Additional file 3). Regarding repressed genes, those with homologs in the Schoggins’s dataset that were downregulated in B57 were not repressed in A22, underscoring the differences of the two responses (Fig. 3).

As an alternative approach to define potential ISG, we also identified, among the list of genes modulated either in B57 or in A22 by the inactivated VHSV, genes with a human homolog in the Interferome database (section type I IFN) [31] (Additional file 4). This analysis identified 128 genes induced more than five times in B57 cells, for only 37 in A22, confirming the trend seen for the Schoggins’s ISG dataset.

Hence, a strong and typical type I IFN response was induced in B57 cells derived from fish resistant to VHSV infection, while cells from the susceptible genetic background A22 up-regulated only a few genes belonging to the IFN related pathway. However, A22 cells did show some transcriptional response implicating genes of the type I IFN pathway. Thus, a few typical markers of this pathway were responding after incubation with the inactivated virus in both A22 and B57, including *irf7*, one *cd9* and one *ddit4*, two *tnfsf10*, and several *map3k5* and *fintrim* genes. Finally, a few genes with IFN-inducible human homologs, were induced in A22 but not in B57, comprising *cyp1B1*, *angPTL1*, a *serpin1* and three *map3K5* genes.

**Fig. 4:**
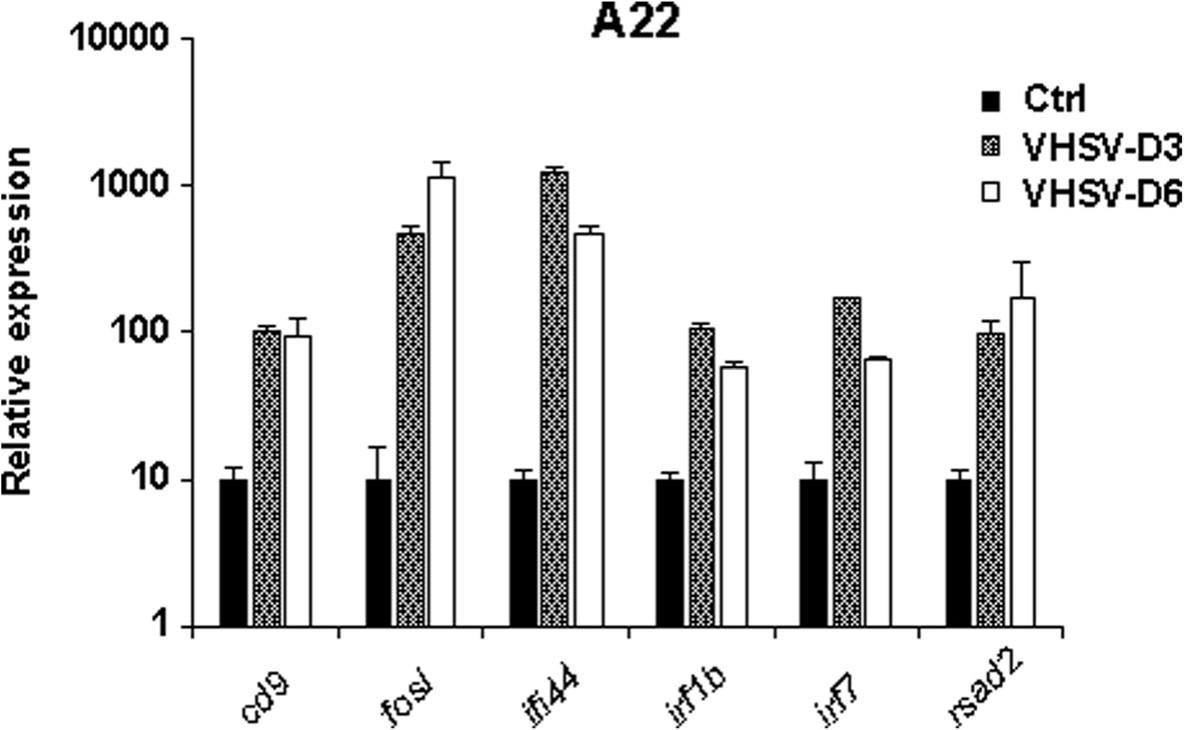
ISG expression is not impaired in A22 fish. A22 fish were infected with VHSV 07–71. Fish were sacrificed at day 3 and 6 post virus inoculation, and RNA was extracted from spleen of infected animals. Gene expression was then assessed by qRT-PCR. Results are expressed as means ± SD relative gene expression compared to uninfected control tissue (Ctrl, set at 10) from three biological replicates (except VHSV D6 – two biological replicates) in technical duplicates

These results raised the question of the respective inducibility of the relevant ISG markers in each genetic background. In particular, were ISG induced in B57 but not in A22 after 24 h incubation with the attenuated virus actually inducible in the latter genetic background? To address this issue, the expression level of a set of typical ISG comprising *ifi44*, *irf7* or *rsad2* was determined in the spleen of A22 fish three and 6 days after VHSV infection by immersion. As shown in Fig. 4, a strong induction of these genes was observed, indicating that their induction by a viral stimulation was not intrinsically impaired in the A22 background.

In summary, it is clear that the A22 response to viral infection is qualitatively and quantitatively different from the one seen in B57, either because genes are modulated (or not) by distinct mechanisms, and/or because the kinetics of the response is different in B57 and A22 cells. In fact, while the total numbers of up- or down- regulated genes are lower in A22 than in B57 cells, the A22-specific response represents more than 840 genes, mostly without obvious connection to antiviral immunity.

### A22 and B57 responses to viral stimulation are not globally correlated to the difference in the steady state level of gene expression

We then performed an analysis of transcriptome of A22 and B57 cells at the steady state to better understand the context of their response to the virus. The genes with significantly different basal expression levels in the two genetic backgrounds were identified and are listed in Additional file 5. Out of about 15,000 genes accurately measured, we found 1692 genes more expressed in B57 [0.4 < FC < 2.5; adj *p*val < 0.01], while 1425 genes were preferentially expressed in A22. Overall, 25% of these 4117 genes differentially expressed between A22 and B57 at steady state were either up- or down- regulated after viral stimulation in at least one cell line (i.e., 1042 genes out of a total of 4238 genes up or down regulated by the viral stimulation either in A22 or in B57). No clear correlation was observed between FC in A22 or in B57 treated with VHVS and the ratio A22/B57 in naive controls; also, no pattern of distribution in the response to VHSV was detected between A22 and B57 up- or down- regulated genes either. The only general trend was that genes highly expressed in a given genetic background generally show slightly lower FC in the response to the viral stimulation.

### Naive B57 cells exhibits higher expression of Jak/STAT-related genes than A22 cells

We then looked for specific functional modules that were differentially expressed in steady state B57 and A22 transcriptomes, and might be important for the resistance status. As shown in Table 3, a functional annotation of genes overexpressed in naive B57 cells compared to A22 cells pointed the Jak/STAT signaling pathway, based on the homologs of *jak1*, *jak2b*, *stat1*, and *irf9* that are implicated in the Jak/STAT pathway activation. This may explain, at least partly, the ability of B57 cells to mount a faster and more efficient IFN response after virus infection compared to A22 cells.

**Table 3:** DR pathways modulated in B57 or A22 genetic background

Induced in B57		Induced in A22	
*1692 RT sequences / 1082 DR genes*		*2425 RT sequences / 1399 DR genes*	
KEGG pathway	*p*value	KEGG pathway	*p*value
Lysosome	0.01	Focal adhesion	0.00003
Ether lipid metabolism	0.01	Regulation of actin cytoskeleton	0.00004
Phosphatidylinositol signaling system	0.01	ECM-receptor interaction	0.0001
Inositol phosphate metabolism	0.01	p53 signaling pathway	0.0007
Porphyrin and chlorophyll metabolism	0.03	Tight junction	0.0027
Notch signaling pathway	0.04	MAPK signaling pathway	0.01
Glycerophospholipid metabolism	0.04	Steroid biosynthesis	0.02
Adherens junction	0.05	Vascular smooth muscle contraction	0.02
**Jak-STAT signaling pathway**	0.08	Wnt signaling pathway	0.02
TGF-beta signaling pathway	0.09	Folate biosynthesis	0.04

*DR Danio rerio*

*RT* rainbow trout

**Bold:** Pathways related to antiviral innate immune response

On the other side, genes highly expressed in A22 cells included genes belonging to cell to cell adhesion, focal adhesion and tight junctions, including the homolog of the *fna1* gene. As fibronectin (*fn1a*) was described as the first VHSV receptor at the cell surface of rainbow trout cells [32], the higher rexpression of this receptor might actually contribute to the susceptibility to viral infection observed in A22. Notably, the MAPK pathway (which includes kinases involved in inflammation and cell proliferation) was also enriched among genes highly expressed in A22 cells, suggesting that this activation pathway may be pre-set in this background (Table 3) in a way that would not favor its contribution to IFN signaling.

### Genes with top contrasted expression between A22 and B57 after viral stimulation point to several ISGs

We then combined the comparisons between A22 and B57 transcriptomes at steady state, and between control and stimulated cells for each background, to identify genes with the largest difference of absolute expression level. We considered the parameter r defined as follows, to translate at best the contrast of expression in B57 and A22, between stimulated cells and steady state:

$$ r=\frac{FC\left( viral\ stimulation\right)\  in\ B57}{FC\left( viral\ stimulation\right)\  in\ A22}\times \frac{expression\ in\ B57\  at\  steady\ state\ }{expression\ in\ A22\  at\  steady\ state\ } $$

r is defined only for FC with adjusted *p* values < 0.01.

Values of r are given in Additional file 3. Five ISG were among genes with the highest value of r: i.e. *dhx58, irf1, cd9, ogfr, and cyp1B1.* Several *fintrim* proteins were also highlighted by this analysis for high (a *btr/trim39* like GSONMG00054800001 and a *Trim35* like GSONMG00023727001) or low (a *MIDLINE1* homolog, GSONMG00073000001) r values. An homolog of *RPT3*, encoding a chemosensory receptor transporter protein, was expressed forty times more in B57 than in A22 at steady state, and was induced 16 times in B57 but only 4 times in A22, leading to a r value of 168. Strikingly, this gene is a paralog of the ISG *RTP4*, and was among the most strongly up-regulated genes by VHSV infection in the whole trout larvae at first feeding and 3 weeks later in our previous study [33]. In this case, fish were from the INRA genetically diverse trout strain “INRA-SY”, indicating that the strong up-regulation of *rtp3* is not a particularity of the A22/B57 isogenic lines.

### The QTL area contains several genes involved in antiviral innate immunity but no DEG

While genes with highly contrasted expression in A22 and B57 cells following the viral stimulation may act downstream of the genetic switch determining the resistance, they constitute potentially interesting candidates for studies of antiviral mechanisms as conspicuous outliers in the response to the infection.

To investigate whether such genes might be located in the genomic region containing the QTL of resistance to VHSV, DEG exhibiting high values of the B57/A22 ratio (*r* > 19) (Additional file 3) were mapped on the rainbow trout genome assembly (GCA_002163495.1; 2017) [34]. The top induced or repressed genes in B57 compared to A22 at the basal level were also mapped, (A22/B57 < 0.015 or > 110, respectively, (Additional file 5)), as well as ISGs specifically up-regulated in B57 after virus stimulation. As shown in Additional file 6, none of these genes are located in the telomeric region of chromosome 3 close to the QTL region. In keeping with this, while the *fn1* gene encoding the fibronectin 1 which is a VHSV receptor [32] was overexpressed in A22 compared to B57, it was found on chromosome 7 and cannot represent the primary explanation of the lack of resistance linked to A22 genetic background. To complete this exploratory survey, we also mapped 20 additional key factors of the type I IFN response. Interestingly, several cytokine receptors, such as *crfb4* and *ifnar1b* as well as *tlr7* and *LOC110520614/tlr8a1* (which were previously characterized as encoding the RNA sensors Toll-like receptors 7 and 8, respectively [35]) were found close to the expected QTL peak (less than 1 Mb from the markers closest to the QTL peak, ie S0172/483H06 andOmy1392INRA, see Additional file 7). A gene encoding a Complement C1q-like protein, orthologous to another typical ISG, was also detected in this region, but out of the QTL area (Additional file 7). These observations indicate that a number of important players of the type I IFN response are encoded by genes located in the neighborhood of the QTL, calling for further functional characterization of the gene content of this region.

## Discussion

In this work, we combined two complementary approaches of quantitative genetics and transcriptome analysis to obtain new insights into the complex mechanisms involved in the resistance of rainbow trout to the rhabdovirus VHSV.

In our previous studies on rainbow trout resistance to VHSV, we identified a major QTL from crosses between resistant and susceptible trout families, which largely explained both fish survival after an immersion challenge with the virus and viral replication in fin explants [13]. We also derived fibroblast-like cell lines from resistant (B57) and susceptible (A22) double-haploid rainbow trout isogenic lines, and observed that their resistance was fully consistent with the one of the parental fish lines [12]. Altogether, these results indicated that intrinsic/innate mechanisms play a major role in resistance.

In order to further investigate the mechanisms involved, we focused here on the two cell lines (A22 and B57) with contrasted resistance. We first applied a QTL approach to these genetic backgrounds to confirm that the difference among the two lines was driven by the same QTL (i.e. most likely the same innate/intrinsic mechanism). Moreover, we extended the phenotyping of resistance using immersion vs injection route of infection, and demonstrated that the main resistance mechanisms are not limited to the virus entry into the host but do rely on.

We previously observed an early induction of *ifn1* (a fish type I IFN also known as *ifnφ1*) by VHSV infection in the resistant cell line B57, but not in the A22 susceptible cells [12]. This observation suggested a possible role of the type I IFN response in resistance, but in the context of a strong difference of viral replication between susceptible and resistant cells. To normalize the stimulation conditions, we used here a virus inactivated by β-propiolactone. Our transcriptome-wide RNAseq study confirmed a drastic difference of type IFN response 24 h post viral stimulation: more than 60 ISG were modulated in B57 only, while seven ISG were modulated only in A22. This difference was not due to an intrinsic defect of the IFN or ISG in A22, which would suppress their induction: in infected A22 fish, where the virus replicates quickly, these genes get strongly up-regulated 3 days post infection. Hence, it rather appears that the sensing, signaling, or amplifying layers of the type I IFN response, potentially affecting its speed and/or its amplitude, are different between B57 and A22 cells, and leads to the contrasted response observed here. As the structure of the IFN pathway is complex, with many positive feedback loops at multiple levels, it is difficult at this stage to identify the defective factors. Further QTL approaches using advanced backcrosses and/or additional families (e.g. GWAS approaches) to precise the QTL region and to identify the key causing gene would help to address this issue. Interestingly, the global transcriptome response - not only the IFN response - was overall weaker in A22 cells, which might be linked to a lower capacity to respond - directly or indirectly - to the VHSV stimulation beyond the IFN pathway. However, the A22 response was still significant with more than 1700 genes up- or down- regulated by the virus stimulation. Importantly, genes induced in both A22 and B57 at comparable levels, should be less (or not) affected by the genetic differences discussed above. Among those genes are a few typical ISGs playing well-defined roles in the antiviral response, such as *irf7* or *cd9*. The functions of others remains undefined in the context of the resistance to VHSV. The case of *fintrim* [16, 36] is particularly interesting: discovered as VHSV- and IFN- induced genes in rainbow trout leukocytes [16], they display signatures of positive selection suggesting that they may be involved in the antiviral response [36]. However, a direct IFN-dependent antiviral activity has been shown for only one member of this large gene family in zebrafish [37], and it is not clear whether all *fintrim* are actually involved in the IFN response. Our observations also suggest that a number of *fintrim*, which are induced similarly in A22 and B57, may play a role in the antiviral defense independently of the IFN response, a possible strategy to counteract viral subversion strategies. Further investigations will be necessary to understand the diversity of the antiviral mechanisms mediated by these highly diversified factors [37].

A differential regulation of the type I IFN response in cells resistant and susceptible to VHSV might suggest a very general mechanism, potentially common to many viruses. This is not the case since the VHSV-resistant B57 line is in fact quite susceptible to a related novirhaboviruses, the Infectious Hematopoietic Necrosis Virus (IHNV) [38]. In fact, the subtle mechanisms of pathogen-sensing and IFN signaling differ markedly between viruses, with multiple systems of viral subversion. Hence, most resistance mechanisms are likely to be specific for a virus - or a small group of related viruses.

Regarding the genetic determinism of the resistance to VHSV, our study bridges a gap between our primary QTL description [13] and the characterization of the response in fibroblastic resistant and susceptible cell lines to viral stimulation. First, our data showed the presence of a major QTL for fish resistance to VHSV between B57 (resistant) and A22 (susceptible) genetic backgrounds, which matched exactly with the one we identified previously in other genetic backgrounds [13]. This observation validated the importance of this region for the resistance to VHSV in multiple haplotypes. Furthermore, our data clarified the type of mechanisms corresponding to the QTL for resistance to VHSV. The QTL had been initially defined for a waterborne VHSV infection, fish injected intraperitoneally showing very high level of mortality, whatever their QTL haplotype [9]. Hence, the resistance appeared somewhat linked to a possible blockage of virus entry into the host. We showed here that the QTL is also relevant for the severity of the disease after virus injection, indicating that underlying resistance mechanisms are actually active during VHSV propagation in the host, but may not be sufficient to block the infection after an intraperitoneal injection. This view is also well consistent with our previous data from cell lines [12], which suggested an innate/intrinsic mechanism potentially active in all cells and likely not restricted to entry sites in fins [32], or possibly in skin or mucosa. The presence of this major QTL needs to be further confirmed in different populations, particularly in the different isogenic lines exhibiting contrasted resistance to VHSV we previously described [9] to generalize its importance for the resistance to VHSV across all rainbow trout genotypes. At this stage, the presence of this QTL and the importance of its effect in outbred commercial populations of rainbow trout still have to be evaluated to determine whether this QTL could be used for marker assisted selection of naturally resistant animals in a breeding program.

Besides unifying observations made in vivo after VHSV infection via two routes and results from in vitro experiments, our data also define for the first time the limits of the QTL within a region bounded by Omy1241INRA and OMM1599 (Fig. 1). The QTL was first described in gynogenetic double-haploid F2 individuals obtained from a F1 female and localized in the telomeric region of Omy3 [13]. However, the low recombination rate in this region during female meiosis, did not allow to discriminate the position of the different markers of the area and to define the limits of the QTL confidence interval [39]. In this study, the presence of the QTL was confirmed by backcrossing a F1 male with a homozygote female. Thanks to the higher recombination in the telomeric regions of the chromosomes during the male meiosis, it was possible to segregate the locations of the different markers on the chromosome, and thereby to refine the position of the QTL and identify the bounded markers.

The more accurate localization of the QTL area and the progress of the rainbow trout Omyk_1 genome assembly ([34], GCA_002163495.1) opened the door to the analysis of the gene content of the main region determining the resistance to VHSV. Whereas none of the DEG identified in these study were found in the predicted QTL, *tlr7* and *LOC110520614/tlr8a1*, which encode the Toll-like receptors 7 and 8 (TLR7 and TLR8) are located in the telomeric region of chromosome 3 [35] (Additional file 6), very close to Omy1392INRA marker (Additional file 7). While the expression of these positional candidate genes is not modulated after VHSV inoculation regardless the genetic background (Additional file 7), a functional mutation in the sequence of these key regulators of the IFN response may explain the observed differences in both susceptibility to VHSV and expression of innate immune genes in resistant and susceptible lines. Indeed, it has been suggested that impaired *tlr7* response might lead to a higher susceptibility to VHSV in Olive flounder (*Paralichthys olivaceus*) [40]. The implication of genes located in this region and their connection to the QTL remain to be demonstrated. Additionally, our analysis did not address modification of the microRNA repertoire, or the possible bias in splicing induced during the response to the virus, which may contribute to differences in susceptibility to VHSV.

## Conclusions

In conclusion, our results improve our knowledge in interactions between rainbow trout and VHSV and open the door to the understanding of the natural resistance to viral infection in fish. By combining a transcriptomic and a genetic approach, we confirmed the relevance of innate immune factors in the resistance to VHSV. The understanding and quantification of the QTL effect would pave the way to the development of attractive selective breeding protocols - where marker assisted selection is advantageous – against VHSV, a major threat in rainbow trout commercial populations.

## Methods

### QTL analysis

#### Experimental family design

F0 breeders come from the previously described INRA rainbow trout isogenic lines [9]. B57 and A22 were selected as resistant and susceptible lines to VHSV 07–71 infection. A B57 female was crossed with a hormonally sex-reversed A22 male to produce the F1 hybrid progeny B57-A22. A hormonally sex-reversed B57-A22 male was then back-crossed with a B57 female to produce the segregating F2 progeny (BCB57-A22) used for QTL detection.

#### Waterborne infection, genotyping and QTL detection

Waterborne challenges using BCB57-A22 juveniles and the VHSV strain 07–71 (serotype 1) were previously described [10]. Two aquaria of 119 and 120 fish were infected. Dead fish were removed twice a day, surviving fish were sacrificed at the end of the challenge (day 39 post-infection), and DNA from every individual was extracted as described [13]. Fish were then genotyped for 8 markers (OMM1053, OMM5005, Omy1009INRA, S0172/483H06, Omy1392INRA, InfGr2int4, and OMM1599) localized in the area of the VHSV major QTL on Omy3 (RT31 linkage group) as described [13]. QTL detection was performed using QTLMap software with a Cox model-based method as described [13, 41, 42]. The 95% confidence interval of the QTL was obtained using the Hendge Li (HL) method [43]. The QTL effect (% of explained phenotypic variance) was estimated by testing the within family effect of the alternative alleles at Omy1392INRA marker (ANOVA with SAS software). QTL effect on survival was estimated using time to death after infection (TTD), assuming a Gaussian distribution and fixing the maximum TTD at day 39.

#### VHSV infection by injection

The infection by intraperitoneal injection was performed with 125 juveniles from the BC57-A22 progeny that were injected with the VHSV 07–71 strain (5000–10 000 PFU per fish) as previously described [9]. Dead fish were removed daily, surviving fish were sacrificed at the end of the challenge at day 28 after infection, and individuals DNAs were extracted as described [13]. Fish were then genotyped for Omy1392INRA, a microsatellite marker close to the most likely position of the VHSV major QTL on Omy3 linkage group.

### Transcriptome deep sequencing analysis

#### Cell lines, virus, and RNA preparation

Fibroblastic cell lines derived from A22 and B57 ovaries were already described [12]. Briefly, cell lines were derived as described for the RTG-2 line: ovary were trypsinised under mild shaking for 2 h, and the cell suspension was cultured at 20 °C in modified Mac Pherson Stoker eagle’s medium supplemented with 10% fetal calf serum, and 100 IU per ml penicillin and 100 μg per ml streptomycin. Several cell lines with typical fibroblast morphology were obtained for each parental fish clone, and displayed similar levels of susceptibility of VHSV for a given genetic background. Cells used in this study have passaged 20 times. VHSV 07–71 strain (genotype 1) was inactivated using β-propiolactone at 1:4000 for 1 h at room temperature. Inactivated virus was then kept at 4 °C for 72 h to eliminate β-propiolactone-induced cytotoxic effects. Cells were inoculated with the inactivated virus (MOI 1) for 24 h. Total RNA was then extracted from three replicates per cell lines using RNeasy Mini Kit (Qiagen) following manufacturer’s instructions.

#### Illumina library preparation and sequencing

The 12 mRNA-Seq libraries were prepared using TruSeq™ RNA Sample Preparation Kit (Illumina) according to the manufacturer’s instructions. Briefly, Poly-A RNA were purified from 4 μg of total RNA using oligo(dT) magnetic beads, fragmented and retro-transcribed using random primers. Complementary-DNAs were end-repaired and 3-adenylated, indexed adapters were then ligated. Ten rounds of PCR amplification were performed and the PCR products were size selected on a 2% agarose E-Gel (Thermo Scientific). Libraries were checked for quality on Agilent High Sensitivity DNA Kit and quantified with the QPCR NGS Library Quantification kit (Agilent Technologies). cDNA libraries were tagged and grouped in nine pools in equal ratios and sequenced in pair-end 2x100bp on Illumina HiSeq2000 with TruSeq™ v3 Kit.

#### Mapping reads and gene expression counts

The read quality was checked with FastQC in the ng6 environment [44]. Reads were then spliced-aligned to the 46,535 genes from the trout reference genome [45] using TopHat v2.0.5 software [46] with the following parameters –r 10 –max-intron-length set to 25,000. The average number of read per sample (R1 + R2) was 90.5 million ±20.3, of which 65.9% ± 0.7 were mapped unambiguously. Secondary mapping (ie, mapping on two or more sites) represented 6.1% ± 0.6 of the total number of reads. All the resulting bam files were merged to produce a unique reference alignment on which the discovery of new genes and transcripts was performed using Cufflinks 2.0.0 [47] with the parameter -l set to 25,000. The resulting GTF file was used as a unique reference for the quantification step of the 12 available samples. The quantification was performed using a locally modified version of cufflinks (sigcufflinks, available upon request on sigenae.org), which produces, per sample, an additional file containing the counts of aligned reads and read pairs. All the count files were merged to produce the final expression table.

#### Identification of differentially expressed genes

Differentially expressed genes between cells treated with the inactivated virus and controls were identified for each cell line. These DEG lists were then compared between A22 and B57. In parallel we also determined DEG between the control A22 and B57, to compare transcriptome differences at steady state, and the DEG between stimulated A22 and stimulated B57 to confirm that the result was similar the analysis of the stimulation within each cell line (data not shown). DEG were identified using DESeq2 1.16 (BioConductor) [48] and R: 3–4-2 [49]. Briefly, raw counts of genes were subjected to a minimal pre-filtering step by removing rows for which the count sum in 12 samples is less than 1. Raw counts were normalized for library size and normalized data were fitted thanks to a negative binomial general linear model. Estimates of Log Fold Changes (LFCs) are shrunken in a manner that removes the problem of exaggerated LFCs for low counts [47], and curbs the risk for erroneous interpretation when using the r parameter as defined above. The shrinkage of LFC estimates can be described as a bias-variance trade-off. Data were adjusted for multiple testing using the Benjamini-Hochberg procedure (adjusted *p*value). The design formula included two factors: the genotype (B57 and A22) and the condition (control versus virus-inoculated). An interaction term (genotype x condition) was added to the formula in order to test if the condition effect differs across genotype. Reference levels for genotype factor and condition factor were set respectively to B57 and to control using the “relevel” function. Genes with an adjusted *p*value less than 0.01 and an absolute Fold Change (FC) > 2.5 or FC < 0.4 were considered as DEGs. Venn diagram were plotted by using Venny 2.1.0 [50].

#### Functional annotation analysis

Rainbow trout protein models [45] were compared to reference zebrafish and human proteomes using blastp (BLAST®, [51]), and the best blast hit annotations were collected. For analysis of multigenic families comprising members induced by the virus, the annotation table was manually curated (for example in Fig. 2). To perform functional annotation analysis of DEG, we used DAVID 6.8 [52, 53] and the zebrafish pathway database in the Kyoto Encyclopedia of Genes and Genomes (KEGG) using DAVID basic parameters (count: minimum 2; *p*value < 0.1). Zebrafish homologs of rainbow trout DEG were used. We are aware that doing so, we lost potentially interested information regarding the expression levels of multiple trout paralogs. Modulated pathways are presented in Table 1 (induced & repressed genes after virus inoculation together), Table 2 (induced genes after virus inoculation only), and Table 3 (induced genes in B57 or in A22 at the basal level). A selection of Rainbow trout genes were mapped on the rainbow trout genome and are presented in Additional file 6.

### Quantification of ISG expression in A22 fish

Waterborne challenges using juveniles and the VHSV strain 07–71 (serotype 1) were previously described [10]. Five VHSV-infected A22 fish and six non-infected control fish were used. Three fish were sacrificed at day 3 post infection, and the two remaining animals were sacrificed at day 6 post-infection. Total RNA was extracted from the spleen using RNeasy Mini Kit (Qiagen). RNA were reverse transcribed into cDNA using SuperscriptII reverse transcriptase (Fischer Scientific) and gene expression was assessed by qPCR using a CFX96 thermocycler (BioRad) following manufacturer’s instruction. Primers used for the detection of rainbow trout *cd9*, *fosl*, *ifi44*, *irf1b*, *irf7*, and *rsad2* are presented in Table 4. All gene expressions were normalized to *actin* expression. QPCR primers were designed using Primer3, and validated for target specificity and for an amplification efficiency > 80%.

**Table 4:** List of primers used for qRT-CPR quantification of rainbow trout genes

*cd9*	Forward	CGCTCAGTATGTGGACAAAGG
	Reverse	CGCATTGGACTCAAAGAGGT
*fosl*	Forward	CTCAGGGCTTCCCACATTAG
	Reverse	ACGGTAAAGGATGTCACAGGA
*ifi44*	Forward	GCTGCACTGAGGAACTTTGA
	Reverse	ACCAGGGCATTATGTGTTGA
*irfb1*	Forward	GTCCAGAGAGCCAATGGAGT
	Reverse	GTCGGTGTAAGGGATGTCGT
*irf7*	Forward	ACCAAGCGTTTCATGATGGT
	Reverse	TCCTCTATGTGTGGGCTTCC
*rsad2*	Forward	GCAACTCCAAGCAGTGTCAA
	Reverse	AAACCTCTCTTTGCTTCCTCAA
*actin*	Forward	GGTGGTACGGCCAGAGGC
	Reverse	GGGAGAAGATGACCCAGATCATG

## Additional files

Additional file 1: Similar transcriptional response dynamics in B57 and A22 cells. The distribution of the fold change of all induced genes, or only ISG as defined for Fig. 2, was represented across the basal level of gene expression. (PDF 360 kb)

Additional file 2: DR pathways modulated in trout cell lines after inoculation with VHSV. (XLSX 10 kb)

Additional file 3: Description of genes significantly up- or down- regulated by incubation with attenuated VHSV in B57 or A22 cells (adj. *p* < 0.01, FC > 2,5 or < 0.4). (XLSX 1214 kb)

Additional file 4: List of rainbow trout genes significantly induced after incubation with inactivated VHSV (adj. *p* < 0.01, FC > 2,5 or < 0.4), having human putative homolog(s) registered in the Interferome database (Section type I IFN) [http://interferome.org/interferome/home.jspx]. (XLSX 208 kb)

Additional file 5: Description of genes significantly up- or down- regulated in B57 naïve cells compared to A22 naïve cells. (XLSX 878 kb)

Additional file 6: Mapping of DEG and innate immune-related genes on the rainbow trout reference genome. (XLSX 17 kb)

Additional file 7: Mapping of candidates genes and microsatellite**/**SNP markers on the two versions of the rainbow trout genome. (XLSX 11 kb)

## Abbreviations

DEG: differentially expressed genes
FC: fold change
IFN: interferon
IHNV: Infectious Hematopoietic Necrosis Virus
IPNV: infectious pancreatic necrosis virus
ISG: Interferon Stimulated genes
KEGG: Kyoto Encyclopedia of Genes and Genomes
NV: non virion non-structural protein
QTL: major quantitative trait locus
RNAseq: RNA sequencing
VHSV: viral hemorrhagic septicemia virus
