# Supplementary figures and images for: Genetic and transcriptomic analyses provide new insights on the early antiviral response to VHSV in resistant and susceptible rainbow trout
Source: BMC Genomics. 2018 Jun 19;19:482. doi: 10.1186/s12864-018-4860-1 (PMC6009034; doi:10.1186/s12864-018-4860-1)

Additional file 1. Similar transcriptional response dynamics in A22 and B57 cells

B57

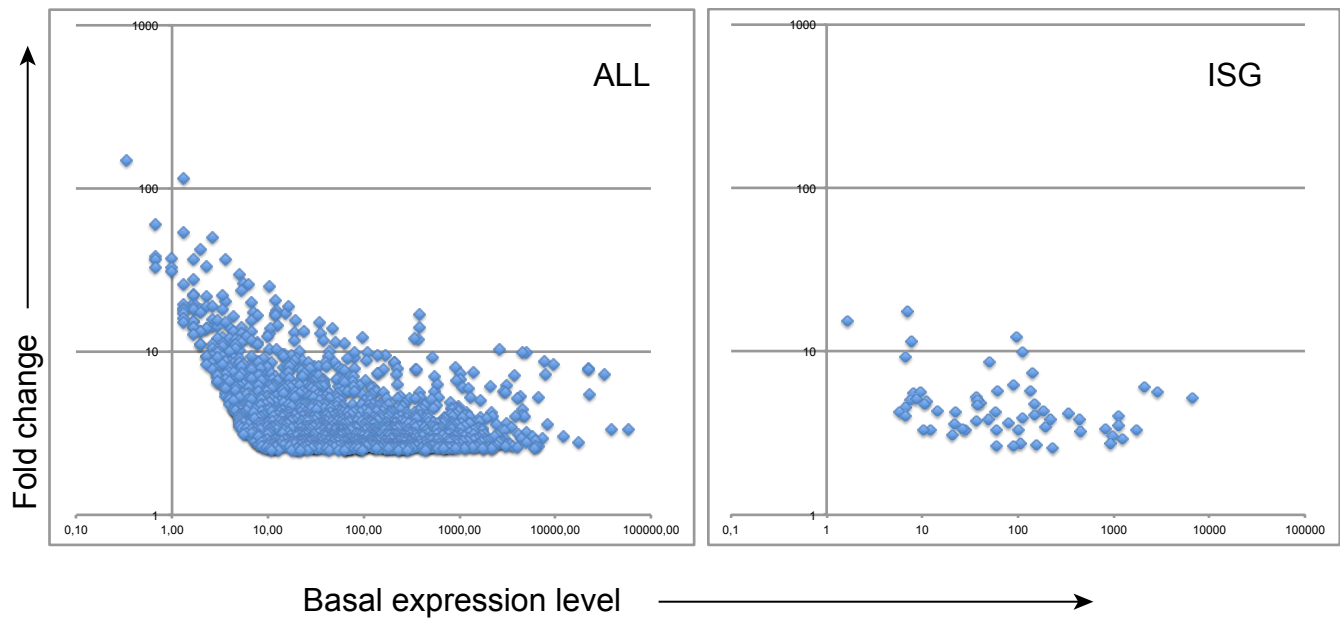

A22

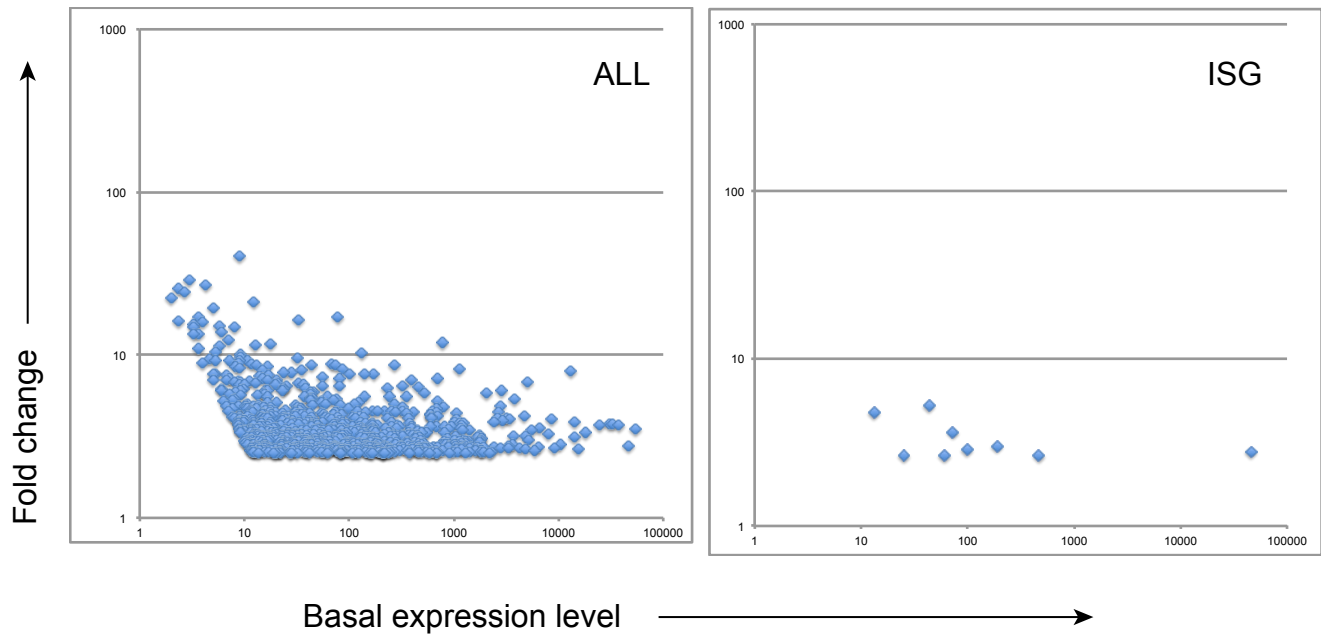

Supplement: Supplementary file 1 — Similar transcriptional response dynamics in B57 and A22 cells. The distribution of the fold change of all induced genes, or only ISG as defined for Fig. 2, was represented across the basal level of gene expression. (PDF 360 kb) [file 12864_2018_4860_MOESM1_ESM.pdf]
